# Comprehensive profiling of phytochemical compounds, antioxidant activities, anti-HepG2 cell proliferation, and cholinesterase inhibitory potential of *Elaeagnus mollis* leaf extracts

**DOI:** 10.1371/journal.pone.0239497

**Published:** 2020-09-23

**Authors:** Jingmiao Li, Yao Ma, Lijuan Kong, Yulin Liu

**Affiliations:** College of Forestry, Northwest A&F University, Yangling, Shaanxi, China; Institute for Biological Research "S. Stanković", University of Belgrade, SERBIA

## Abstract

The aim of this work was to enrich the knowledge on the potential applications of *Elaeagnus mollis* leaf extracts. For this purpose, the bioactive compounds (phenolic, flavonoid, alkaloid, proanthocyanidin, chlorophyll and carotene content), antioxidant activity, anti-HepG2 cell proliferation, and cholinesterase inhibitory potential (AChE and BChE) of *E*. *mollis* leaves which obtained from different habitats were quantitatively analyzed using various solvents (water, methanol, ethanol, and n-hexane). The results showed that the methanol extracts exhibited the strongest 1,1-diphenyl-2-picrylhydrazyl (DPPH) free radical scavenging activity and the water extracts showed the best antioxidant activity in the 2,2’-azinobis-3-ethylbenzothiazoline-6-sulfonic acid (ABTS) free radical scavenging activity, ferric reducing antioxidant power (FRAP), and reducing power (RP) assays. Moreover, the methanol extracts showed the best inhibitory activity against cholinesterase and HepG2 cancer cells. Correlation analysis revealed that the high antioxidant and anti-HepG2 cell proliferation activities were mainly attributed to the total phenolics, flavonoids, and proanthocyanidins while AChE inhibition was attributed to the total alkaloid and carotene content. The statistical results showed that the effect of habitats was lower than that of different solvents used. Additionally, the metabolic profiles of *E*. *mollis* leaves were evaluated using HPLC-ESI-Q TRAP-MS/MS, and a total of 1,017 chemical components were detected and classified into 23 classes. The organic acids and derivatives ranked the first, followed by flavone, amino acid and derivatives, and so on. In conclusion, the effects of different solvents were more significant than the effects of different habitats and the methanol extracts of *E*. *mollis* leaves could be used as an effective source of functional active components, provide benefits to physical health care and be applied to the food and pharmaceutical industries.

## Introduction

Various natural compounds have been demonstrated to play an important role in ameliorating the development of diseases that have a contact with oxidative stress, i.e., cancers, neurodegenerative diseases, cardiovascular diseases and inflammation [1–3]. Thus, it is well accepted that natural compounds, including flavonoids, phenolics, alkaloids, proanthocyanidins, chlorophyll, carotene, etc. found in different plant tissues are powerful antioxidants and have attracted increasing attention and study.

*Elaeagnus mollis*, from the family Elaeagnaceae, is a surviving plant from the unique paleontological tree of Quaternary glaciation in China. It is regarded as an economic plant that is cultivated widely due to its strong environmental adaptability, with seeds that are rich in good quality edible oil [4]. Until now, the focus of some studied has been on the seeds of *E*. *mollis*, and have concerned their nutrient content, active constituents, and medical benefits [5, 6], while comprehensive evaluation of the chemical components, bioactive compounds, and biological activity of the leaves has been scarce, although the leaves of *E*. *mollis* have been used for herbal tea by the local population for many years [6].

In general, the constituents of plants are closely related to the location, soil conditions, and species [7, 8]. In addition, they are also affected by the extraction solvent [9], temperature [10], and technique [11]. Among these factors, the nature of the solvent and habitat of the plant are considered the most critical. Several previous studies have shown that the total alkaloids and polysaccharides contained in *E*. *mollis* leaves exhibit strong free radical scavenging activities [12, 13]. However, these studies usually used a single solvent for extraction, isolation, and detection without considering the influence of different solvents and habitats on the bioactive compounds and biological activity, and there has not been enough comprehensive research in this field so far.

In this study, we focused on the effect of different solvents and various habitats on the bioactive compounds and biological activities of *E*. *mollis* leaves with the aim of evaluating their potential applications. The bioactive compound content, antioxidant activity, anti-HepG2 cell proliferation activity, and cholinesterase (ChE) inhibition of the leaves extracted with different solvents and obtained from different habitats are comprehensively reported herein. These results provide new information for the potential use of *E*. *mollis* leaves as a natural source for healthcare, pharmaceutical, and food processing applications.

## Materials and methods

### Reagents

All reagents used in the extractions were of analytical grade. Methanol, ethanol, n-hexane, hydrochloric acid, chloroform, anhydrous sodium carbonate, aluminum nitrate, Tris-HCl buffer, potassium ferricyanide, ferric chloride, ferrous sulfate, sodium nitrite, trichloroacetic acid, potassium persulfate, potassium ferrocyanide, and phosphate-buffered saline (PBS) were purchased from Sinopharm Chemical Reagent Co., Ltd, (Shanghai, China). Ascorbic acid, vanillin, catechin, gallic acid, chelerythrine, quercetin, Folin-Ciocalteu’s reagent, acetylthiocholine iodine (ATCI), s-butyrylthiocholine iodine (BTCI), and 5,5-dithiobis- (2-nitrobenzoic acid) (DTNB) were purchased from Solarbio Technology Co., Ltd. (Beijing, China)

### Plant extract preparation

*E*. *mollis* leaves were collected from four different habitats: Shanxi Agricultural University (SXAU), Yicheng (YC), Northwest A&F University (NAFU), and Xi'an Botanical Garden (BG) in mid-July 2019, and identified by Dr. Xianyun Mu from the School of Ecology and Nature Conservation, Beijing Forestry University (Table 1). No specific permissions were required for these locations/activities, as there are no rare and protected plants around the trees. Our collection has no effect on the local ecological environment. Afterward, the samples were dried, crushed to a fine powder (40 mesh) when necessary, and homogenized. Then, 0.2 g of each powder sample was added to 8 mL of solvent (either water, methanol, ethanol, or n-hexane). Mixtures were subjected to microwave oscillation conditions at room temperature (RT) for 30 min to speed up the extraction process and then allowed to stand for 24 h. The supernatant was filtered to acquire liquid extracts, subsequent calculations of bioactive compound content determination are based on this concentration. And 1 mL of each extract was decanted and evaporated to dryness.

**Table 1:** Coordinates of the plant *E*. *mollis* collecting locations in the Provinces Shanxi and Shaanxi.

Code	Province	Locality	Source	Longitude(E)	Latitude(N)
SXAU	Shanxi	Shanxi Agricultural University	Cultivated	112°58′	37°43′
YC	Shanxi	Yicheng	Wild	111°59′	35°36′
NAFU	Shaanxi	Northwest A&F University	Cultivated	108°07′	34°26′
BG	Shaanxi	Xi'an Botanical Garden	Cultivated	109°03′	34°21′

### Total phenolic content (TPC)

TPC was determined according to the Folin-Ciocalteu method with slight modifications [14]. First, 50 μL of each extract was added to 100 μL of Folin-Ciocalteu’s reagent (50%). After 2 min at RT, 150 μL of sodium carbonate (20%) was added. After incubation for 2 h in the dark, the absorbance was measured at 765 nm; gallic acid was used as the standard, and the TPC is expressed as mg of gallic acid equivalents per gram of dry weight (mg GAE/g DW).

### Total flavonoid content (TFC)

The TFC of the leaf extracts was assessed via an aluminum chloride colorimetric method [14]. First, 25 μL of 5% sodium nitrite solution was added to 50 μL of extract, after which the mixture was allowed to stand at RT for 5 min. Subsequently, 25 μL of 10% aluminum chloride solution was added, and after incubation for 6 min, 200 μL of 4% sodium hydroxide solution was added. The absorbance was measured at 510 nm. Quercetin was chosen as the standard and the results are expressed as mg of quercetin equivalents per gram of dry weight (mg QE/g DW).

### Total proanthocyanidin content (TPrC)

TPrC was determined via the vanillin-hydrochloric acid method [15] with a slight improvement. Briefly, 0.0625 g of vanillin was mixed with 4 mL of hydrochloric acid and 100 mL of methanol, which was then stored at 4°C after mixing thoroughly. For each extract, 180 μL of the above mixture solution was added to 50 μL of extract at RT. Catechin was used as the standard, the absorbance of the samples was measured at 500 nm, and TPrC is expressed in mg of catechin equivalents per gram of dry weight (mg CE/g DW).

### Total alkaloid content (TAC)

TAC was measured by the acid dye colorimetric method with a slight improvement [16]. First, bromocresol green was dissolved in PBS at pH 4.5, and then 2 mL of the dye was mixed with 2 mL of each extract and 4 mL of chloroform. After mixing thoroughly, the sample was transferred to a separatory funnel and allowed to stand for 2 h at RT. The maximum absorption wavelength of the chloroform layer was determined and the absorbance at this wavelength was measured for chloroform as a blank sample and chelerythrine solution of different concentrations as the standard. The TAC is expressed as mg of chelerythrine equivalents per gram of dry weight (mg ChE/g DW).

### Total chlorophyll content (TChC) and total carotene content (TCaC)

TChC and TCaC were analyzed according to the method described by Gunathilake & Ranaweera [17]. The absorbance of each prepared samples was measured at 470, 653, and 666 nm. The chlorophyll a, chlorophyll b, and carotene content values were respectively calculated according to the following formulas, respectively:

$$C_{a}=11.75\;\left(A662\right)-2.350\left(A645\right)$$ (1)

$$C_{b}=18.61\;\left(A645\right)-3.960\left(A662\right)$$ (2)

$$Carotene=1000\;\left(A470\right)-2.270\;\left(C_{a}\right)-81.4\;\left(C_{b}\right)/227$$ (3)

where C_a_ means chlorophyll a content and C_b_ means chlorophyll b content.

### Antioxidant activity

#### 1,1-Diphenyl-2-picrylhydrazyl (DPPH) free radical scavenging activity

DPPH free radical scavenging activity was measured as described previously with some modifications [18]. 20 μL of extract (prepared in a series of concentrations) was added to 180 μL of freshly prepared DPPH solution in ethanol. The reaction was allowed to stand at RT in the dark for 30 min and the absorbance was measured at 517 nm. The radical scavenging activity was calculated as

$$DPPH\;radical\;scavenging\;capacity\;\left(\%\right)=\left[\left(A_{0}‐A\right)/A_{0}\right]$$ (4)

where A_0_ and A represent the absorbance of the blank and test samples, respectively. The scavenging activities of the test extracts expressed as IC_50_ values were compared with the standard ascorbic acid.

#### 2,2’-Azinobis-3-ethylbenzothiazoline-6-sulfonic acid (ABTS) radical scavenging activity

The ABTS free radical scavenging potential of the extracts was determined by a previously reported method [19]. First, the ABTS solution was prepared by the reaction between ABTS (7 mM) and potassium persulfate (2.45 mM) and then stored in the dark at RT for 12 h. The ABTS solution was then diluted to an absorbance of approximately 0.7 at 734 nm. The extracts were serially diluted, 20 μL of each dilution was mixed with 180 μL of ABTS solution, and the absorbance was measured at 734 nm after 6 min. The IC_50_ value was then obtained from the standard curve.

#### Reducing power (RP) activity

The reducing power activity of the extracts was carried out using a previously reported method [20]. 1 mL of extract was added to 2.5 mL of PBS (0.2 M, pH 6.6) and 1.5 mL of 1% potassium ferricyanide solution. After incubating for 20 min, 1.5 mL of 10% trichloroacetic acid was added to terminate the reaction. Then, the solution was centrifuged at 4,000 rpm for 10 min, after which 4 mL of the supernatant was mixed with 0.5 mL of 0.1% ferric chloride solution. Finally, after 1 min, the absorbance was measured at 700 nm, and ascorbic acid was used as a positive control.

#### Ferric reducing antioxidant power (FRAP) activity

FRAP activity was determined using the standard method with a slight improvement [21]. A FRAP working solution was prepared as 1:10:1 (v/v/v) Fe^3+^-TPTZ (10 mM) in 0.04 M hydrochloric acid, 0.3 M acetate buffer (pH 3.5), and 10 mM ferric chloride solution. 15 μL of extract was mixed with 20 μL of freshly prepared FRAP working solution and the absorbance was measured at 593 nm after 3 min, with ferrous sulfate as the standard.

### Anti-HepG2 cell proliferation assay

HepG2 cells at 5 × 10^4^ cells/well were cultured in 1 mL of Dulbecco's modified eagle medium (DMEM) containing 10% fetal bovine serum (FBS) and 100 μg/mL streptomycin/penicillin in 24-well plates at 37°C for 12 h. Afterward, 100 μL of the cells were seeded on a 96-well plate (7 × 10^3^ cells/well). Next, the culture solutions were removed, 100 μL of serially diluted leaf extract was added, and the mixture was incubated for 1 day. Cells in DMEM were chosen as the blank control. Subsequently, the cells were washed three times with PBS, and 20 μL of 3-(4,5-dimethyl-2-thiazolyl)-2,5-diphenyl-2-H-tetrazolium bromide (MTT) (0.5 mg/mL) was added. After the incubation for 1 h, 100 μL of dimethyl sulfoxide (DMSO) was used to dissolve the formazan crystals formed by the active cells and the absorbance of the mixture was measured at 450 nm.

### ChE inhibition assay

The ChE inhibition activity against acetylcholinesterase (AChE) and butyrylcholinesterase (BChE) was measured using reported assay with some modifications [22]. The reaction mixture was composed of 2 μL of extracts (diluted in series), 10 μL of Tris-HCl buffer (50 mM, pH 8.0), 20 μL of ATCh or BTCh (1.5 mM), 100 μL of DTNB (0.3mM), and 20 μL of AChE or BChE solution (0.5 units/mL). The absorbance was measured at 412 nm after incubation at 25°C for 20 min. Huperzine was used as the positive control and as the negative control without the ChE inhibitor.

### High performance liquid chromatography (HPLC)-electrospray ionization (ESI)-Q TRAP-mass spectrometry (MS)/MS assay

The widely targeting metabolomics approach was used to obtain the metabolic profiles of mixed samples from different habitats. The components were identified from the self-compiled metabolite database MWDB of Metware Biotechnology Co., Ltd. (www.metware.cn; Wuhan, China). HPLC-ESI-Q TRAP-MS/MS assays and metabolite data analysis were conducted as previously described [23, 24].

### Statistical analysis

All experimental measurements were performed in triplicate and the data were analyzed with SPSS and the mean ± standard deviation (SD) of the assay results were recorded. Duncan’s multiple comparison test was used to evaluate the differences among the bioactive compounds of the tested extracts; p-values of less than 0.05 were considered statistically significant. In addition, Spearman’s rank correlation analysis was conducted on the total bioactive compound content, antioxidant activity, ChE inhibition, and anti-HepG2 cell proliferation activity of the samples.

## Results and discussion

### Extract yield and total bioactive compound content

The total phytochemical compound content and details of the phenolics, flavonoids, proanthocyanidin, alkaloid, chlorophyll, and carotene contents of *E*. *mollis* leaves from different habitats extracted with a variety of solvents are reported in Table 2. The yields of the leaf extracts were as follows: water ranked first (3.90%), followed by methanol (2.93%), n-hexane (1.19%), and ethanol (1.18%). Among the leaf extracts from the four habitats, BG gave the highest yield whereas SXAU gave the lowest yield.

**Table 2:** The percent yields and total bioactive compound contents in different extracts of *E*. *mollis*.

Assay	Yields (%)	Phenolics (mg GAE/g DW)	Flavonoids (mg QE/g DW)	Proanthocyanidins (mg CE/g DW)	Alkaloid (mg ChE/g DW)	Chlorophyll (mg/mL)	Carotenes (mg/mL)
Water extracts							
SXAU	3.63	0.37±0.01^bc^	2.14±0.17^ab^	97.60±1.49^bcd^	0.10±0.02^g^	5.67±0.35^f^	0.44±0.03^h^
YC	3.63	0.37±0.01^b^	1.96±0.12^bc^	94.63±0.71^cd^	0.15±0.02^f^	6.43±0.89^f^	0.19±0.04^h^
NAFU	3.83	0.34±0.01^cd^	2.10±0.09^ab^	96.81±2.17^cd^	0.10±0.01^g^	6.72±1.20^f^	0.42±0.01^h^
BG	4.50	0.34±0.01^bc^	1.35±0.07^ef^	92.88±2.69^d^	0.12±0.01^fg^	6.48±0.59^f^	0.22±0.01^h^
average	3.90	0.35	1.89	95.48	0.12	6.33	0.32
Methanol extracts							
SXAU	3.10	0.37±0.01^b^	2.28±0.13^a^	121.05±7.91^a^	0.33±0.01^cde^	22.71±1.89^cd^	2.16±0.26^cdef^
YC	2.80	0.48±0.01^a^	2.24±0.03^ab^	123.18±3.26^bcd^	0.37±0.02^abc^	18.07±0.98^e^	1.54±0.28^fg^
NAFU	2.73	0.34±0.01^bcd^	2.15±0.04^ab^	114.23±2.19^ab^	0.31±0.02^cde^	23.86±1.13^c^	1.87±0.12^defg^
BG	3.10	0.32±0.01^d^	1.68±0.22^cd^	108.12±7.51^bc^	0.33±0.03^cde^	43.27±3.80^a^	2.45±0.41^a^
average	2.93	0.38	2.09	116.65	0.33	26.98	2.00
Ethanol extracts							
SXAU	1.07	0.21±0.01^e^	1.64±0.02^de^	99.98±0.36^cd^	0.33±0.01^cde^	22.01±0.22^cd^	2.71±0.08^bc^
YC	1.10	0.33±0.04^d^	1.56±0.03^def^	105.48±4.06^bcd^	0.41±0.01^a^	19.39±1.13^de^	2.50±0.26^cdef^
NAFU	1.20	0.18±0.01^f^	1.30±0.16^f^	100.11±3.15^cd^	0.29±0.00^e^	25.89±0.43^c^	2.35±0.06^ab^
BG	1.33	0.19±0.01^e^	1.25±0.05^f^	106.32±8.95^bcd^	0.36±0.00^bcd^	31.36±0.76^b^	2.89±0.35^cd^
average	1.18	0.23	1.44	102.97	0.35	24.66	2.61
N-hexane extracts							
SXAU	1.13	0.02±0.00^hg^	0.76±0.07^g^	69.85±0.81^e^	0.30±0.02^de^	4.42±0.20^f^	1.58±0.23^efg^
YC	1.13	0.01±0.00^h^	0.58±0.03^gh^	71.11±0.70^e^	0.38±0.02^ab^	6.85±0.53^f^	2.28±0.48^cde^
NAFU	1.23	0.02±0.00^hg^	0.34±0.05^hi^	73.58±1.44^e^	0.33±0.02^bcde^	6.51±0.51^f^	2.40±0.07^a^
BG	1.27	0.05±0.00^g^	0.25±0.03^i^	70.75±1.76^e^	0.34±0.02^bcde^	4.59±0.10^f^	2.09±0.11^g^
average	1.19	0.02	0.48	71.32	0.34	5.59	2.09

Different letters indicate differences in the extracts (p < 0.05).

The phenolic and flavonoid content from the extract were, from highest to lowest, methanol (0.38 mg GAE/g DW and 2.09 mg QE/g DW, respectively), water (0.35 mg GAE/g DW and 1.89 mg QE/g DW, respectively), ethanol (0.23 mg GAE/g DW and 1.44 mg QE/g DW, respectively), and n-hexane (0.02 mg GAE/g DW and 0.48 mg QE/g DW, respectively). Similarly, the order for proanthocyanidin extract content was methanol (116.65 mg CE/g DW), ethanol (102.97 mg CE/g DW), water (95.48 mg CE/g DW), and n-hexane (71.32 mg CE/g DW); for alkaloids, this order was ethanol (0.35 mg ChE/g DW), n-hexane (0.34 mg ChE/g DW), methanol (0.33 mg ChE/g DW) (these first three were almost the same), and water (0.12 mg ChE/g DW); for chlorophyll, this order was methanol (26.98 mg/mL), ethanol (24.66 mg/mL), water (6.33 mg/mL), and n-hexane (5.59 mg/mL); and for carotenes, this order was ethanol (2.61 mg/mL), n-hexane (2.09 mg/mL), methanol (2.00 mg/mL), and water (0.32 mg/mL). It can be concluded that methanol performed better than the other solvents to extract phenolics, flavonoids, proanthocyanidins, and chlorophyll.

The analysis of variance (ANOVA) revealed that the solvent had a significant effect on the extraction of bioactive compounds. It is now understood that the polarity of the solvent plays an important role in the solubility of organic compounds such as phenolics, flavonoids, and so on [25]. Indeed, strongly polar solvents have displayed a higher extraction efficiency for bioactive compounds from plant materials than weakly polar solvents [26]. Similar rules were reported in the case of *Crinum* species [27] and *Thymus numidicus* [18]. There are many factors that affect the extraction efficiency of different active compounds from plants, and the polarity of the solvent is one of them. Bioactive natural substances in plants often have complex structures and connections to each other, so it is difficult to quantify these components separately [28]. This fact may help to explain the inconsistent order between the solvent polarity and the yields, as well as the yields and extraction efficiency of the bioactive compounds.

The solubility of a compound in particular solvents can play an important role in the extraction of the compound, as the process is affected by the properties of the compound and the polarity of the solvent [29, 30]. For example, alkaloids, chlorophyll, and carotenes are insoluble in water but soluble in organic solvents.

There can also be differences in the bioactive compound content in plant extracts from different habitats, indicating that the conditions in different habitats influence the plant bioactive compound content. Previous studies have verified that bioactive compound content may differ depending on the location [31, 32], which can be influenced by various environmental factors, particularly temperature [33]. One possible reason for this result is that temperature may affect the activities of enzymes in the synthesis of related active compounds.

### Antioxidant activities

The antioxidant activity of *E*. *mollis* leaf extracts obtained from different solvents was investigated via DPPH, ABTS, RP, and FRAP assays (Table 3). The results showed significant differences according to the solvent, habitat, and interactions between the solvents and habitats at all levels. In the study, DPPH and ABTS free radical scavenging by the extracts are expressed as IC_50_ values, the lower the IC_50_ value, the higher the free radical scavenging activity [34]. The leaf extract in methanol performed the best in the DPPH assay (13.93 mg/mL), followed by in water (15.05 mg/mL), ethanol (25.56 mg/mL), and n-hexane (52.36 mg/mL). For the ABTS, RP, and FRAP assays, the water extract exhibited the highest activities (3.77 mg/mL, 1142.20 mg/mL and 318.41 mg/mL, respectively), followed by methanol (7.62 mg/mL, 1132.87 mg/mL and 212.69 mg/mL, respectively), ethanol (19.76 mg/mL, 550.26 mg/mL and 103.36 mg/mL, respectively), and n-hexane (115.81 mg/mL, 124.40 mg/mL and 14.57 mg/mL, respectively). Obviously, the results show that higher antioxidant activity was found in the extracts from the more polar solvents, especially water. In addition, the differences in antioxidant activity among the leaf extracts from various solvents and from different habitats can be attributed to differences of bioactive compounds of the extracts. The solvent properties undoubtedly had a great influence on the extraction of antioxidant compounds [35]; polar solvents have a stronger ability to dissolve bioactive compounds than nonpolar solvents, so the polar solvent extracts contained higher levels of bioactive compounds with antioxidant capabilities [18]. Furthermore, the accumulation of bioactive substances in plants from different habitats also varied due to differences in environmental conditions, which ultimately led to differences in antioxidant activity among the leaf extracts from different habitats.

**Table 3:** Antioxidant scavenging activities of various extracts of *E*. *mollis* against DPPH, ABTS, RP, and FRAP.

Assay	DPPH IC_50_ (mg/mL)	ABTS IC_50_ (mg/mL)	RP (mg/mL)	FRAP (mg/mL)
Water extracts				
SXAU	17.28±0.17^d^	3.18±0.08^h^	1653.31±3.53^a^	319.20±2.81^b^
YC	15.43±0.78^de^	3.55±0.08^h^	1287.90±8.67^b^	407.94±7.14^a^
NAFU	15.46±0.58^de^	4.01±0.02^h^	1002.14±5.11^d^	353.87±12.36^b^
BG	12.02±0.42^e^	4.36±0.04^h^	625.43±2.02^fg^	192.65±3.00^d^
average	15.05	3.77	1142.20	318.41
Methanol extracts				
SXAU	13.30±1.06^de^	7.25±0.02^hg^	1136.15±6.75^c^	213.63±2.97^cd^
YC	12.66±1.23^e^	8.56±0.06^fgh^	1170.45±3.08^c^	246.76±8.64^c^
NAFU	15.44±0.91^de^	5.39±0.02^h^	1307.81±1.91^b^	213.22±1.66^cd^
BG	14.32±1.12^de^	9.31±0.16^fgh^	917.05±8.19^e^	177.15±3.12^d^
average	13.93	7.62	1132.87	212.69
Ethanol extracts				
SXAU	26.03±1.11^c^	17.95±0.23^ef^	519.46±5.11^gh^	124.06±3.76^e^
YC	26.40±0.03^c^	23.41±0.27^e^	579.34±6.22^f^	104.47±3.23^ef^
NAFU	25.80±0.57^c^	15.61±1.38^efg^	603.34±4.84^f^	116.51±0.85^ef^
BG	24.03±1.07^dc^	22.08±0.34^e^	498.89±2.64^h^	68.40±4.10^fg^
average	25.56	19.76	550.26	103.36
N-hexane extracts				
SXAU	43.72±0.82^b^	83.55±1.77^d^	180.61±7.55^i^	16.81±1.49^gh^
YC	62.63±0.08^a^	99.43±2.19^c^	119.68±5.17^ij^	10.57±0.16^h^
NAFU	61.75±1.04^a^	150.70±2.00^a^	130.54±0.44^ij^	16.78±1.06^gh^
BG	41.32±0.11^b^	129.57±2.83^b^	66.76±3.31^j^	14.11±1.19^h^
average	52.36	115.81	124.40	14.57

Different letters indicate differences in the extracts (p < 0.05).

### Anti-HepG2 cell proliferation

In recent years, it has been found that plant extracts and natural bioactive compounds from plants show pharmacological activity against different cancers [36, 37]. In the present study, HepG2 cells were selected to assess the anticancer cell proliferation activity of the leaf extracts (Table 4). The methanol extracts performed the best with the lowest IC_50_ value of 0.13 mg/mL, followed by ethanol (an IC_50_ of 0.15 mg/mL), n-hexane (IC_50_ of 0.17 mg/mL), and water (IC_50_ of 0.20 mg/mL). According to the ANOVA results, it revealed that the different solvents had a more significant effect on the anti-HepG2 cell proliferation activity of the extracts compared to the effects of the various habitats. The leaf extract in methanol exhibited the strongest anticancer activity, which could due to the fact that methanol is a strongly polar organic solvent that can extract more bioactive compounds from the leaves. There are several *Elaeagnus* spp. that can be utilized as having potential natural materials that are inhibitory towards cancer cells. For example, an *E*. *pungens* bark extract inhibits SGC-7901 and BEL-7404 cancer cell proliferation [38], and *E*. *angustifolia* extracts inhibit Hela cell proliferation [39], along with other species such as *E*. *bockii* [40] and *E*. *rhamnoides* [41]. Therefore, as a species of the *Elaeagnus* genus, *E*. *mollis* leaf extracts have the potential for use as an anticancer and anti-cell proliferation agent.

**Table 4:** Anti -HepG2 cell proliferation activity of various extracts of *E*. *mollis*.

	Water extracts	Methanol extracts	Ethanol extracts	N-hexane extracts
SXAU	0.23±0.01^a^	0.13±0.01^fg^	0.15±0.01^defg^	0.16±0.01^cdef^
YC	0.17±0.01^bcde^	0.12±0.01^g^	0.14±0.01^efg^	0.17±0.02^bcde^
NAFU	0.19±0.00^bc^	0.13±0.00^fg^	0.16±0.01^cdef^	0.17±0.02^bcde^
BG	0.20±0.01^b^	0.14±0.01^efg^	0.16±0.01^cdef^	0.18±0.00^bcd^
average	0.20	0.13	0.15	0.17

Different letters indicate differences in the extracts (p < 0.05).

### ChE inhibition assay

There are several medicinal plants that have exhibited strong ChE inhibition activity that might be due to the presence of ChE inhibitors [42]. ChE inhibitors are mainly used in the treatment of Alzheimer's disease [43]. Thus, two ChEs (AChE and BChE) were selected to evaluate ChE inhibition in this work. The results show that different solvents, habitats, and their interactions show significant differences (Table 5). The leaf extract in methanol was found to have the highest levels of ChE inhibition, with the mean values of 3.70 mg/mL and 5.48 mg/mL against AChE and BChE, respectively, followed by n-hexane (4.79 mg/mL and 7.34 mg/mL, respectively), ethanol (5.53 mg/mL and 7.49 mg/mL), and water (7.29 mg/mL and 9.19 mg/mL). Among the four habitats, the leaf extracts from *E*. *mollis* grown in NAFU performed the best. In particular, the inhibition of the tested extracts against AChE was stronger than the inhibition of BChE. In previous studies, the fruit and leaf extracts of *Elaeagnus umbellate* were also found to exhibit substantial inhibition activity against AChE and BChE [44]. From the results of the present study, *E*. *mollis* extracts have potential value for ChE inhibition.

**Table 5:** Cholinesterase (ChE) inhibition potential of various extracts of *E*. *mollis*.

Assay	AChE IC_50_ (mg/mL)	BChE IC_50_ (mg/mL)
Water extracts		
SXAU	7.30±0.12^a^	8.45±0.10^d^
YC	7.56±0.02^b^	9.93±0.14^b^
NAFU	7.05±0.10^c^	-
BG	7.23±0.06^bc^	-
average	7.29	9.19
Methanol extracts		
SXAU	3.79±0.04^g^	8.74±0.06^d^
YC	4.56±0.08^f^	6.46±0.10^fg^
NAFU	3.16±0.08^g^	3.62±0.03^j^
BG	3.29±0.06^h^	3.12±0.05^j^
average	3.70	5.48
Ethanol extracts		
SXAU	5.25±0.07^e^	6.33±0.11^gh^
YC	7.31±0.08^bc^	10.66±0.17^a^
NAFU	3.86±0.10^g^	5.48±0.09^i^
BG	5.70±0.09^d^	7.50±0.08^fg^
average	5.53	7.49
N-hexane extracts		
SXAU	4.53±0.07^f^	7.00±0.12^ef^
YC	5.70±0.08^d^	7.22±0.06^e^
NAFU	3.91±0.07^h^	5.86±0.13^hi^
BG	5.00±0.07^d^	9.28±0.12^c^
average	4.79	7.34

Different letters indicate differences in the extracts (p < 0.05);—means no active.

### Correlation analysis

**Fig 1:**
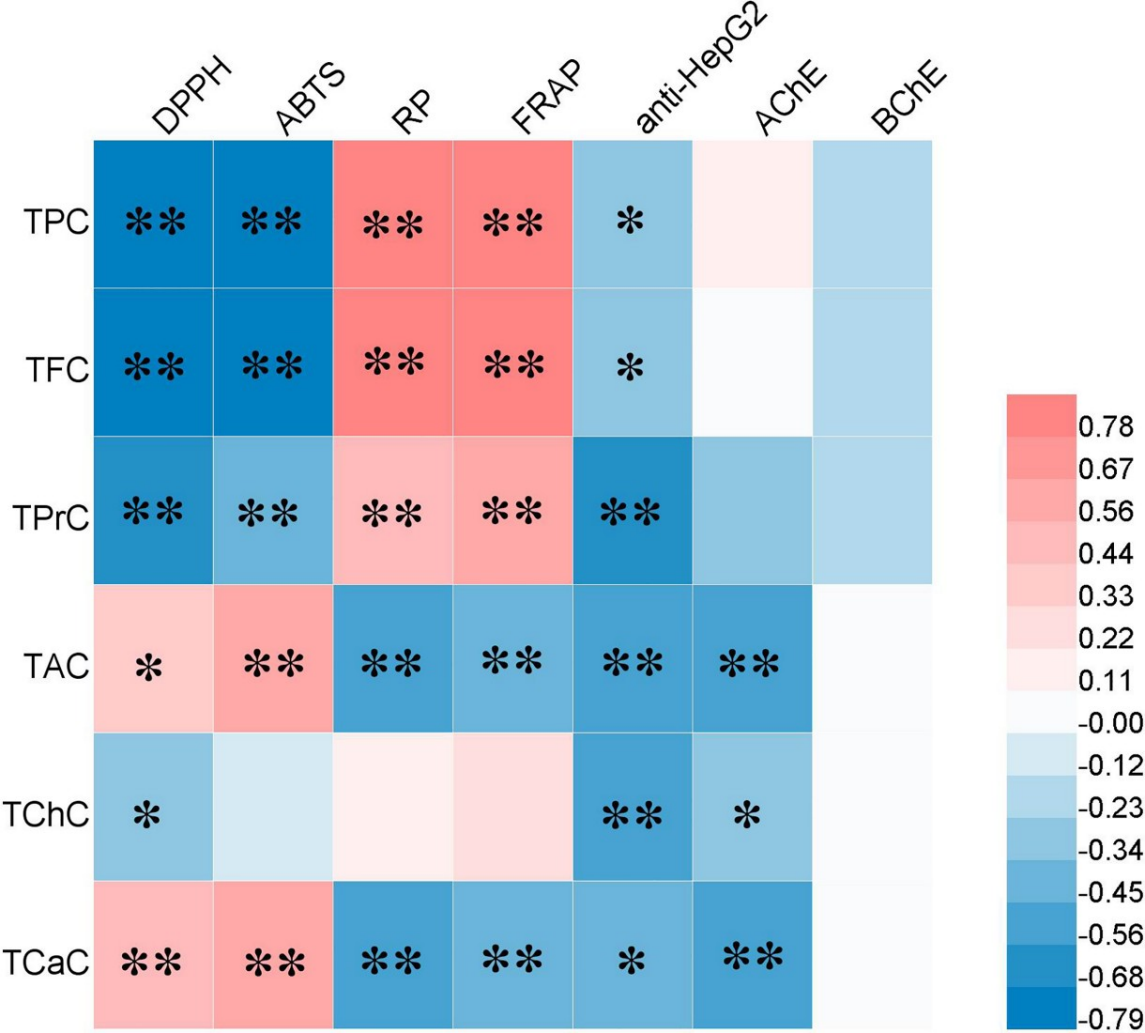
Spearman correlation coefficient between the total bioactive compound contents (n = 16) and antioxidant capacity, cholinesterase inhibition and cytotoxic activity (n = 16). * means p < 0.05; ** means p < 0.01.

Based on the correlation analysis between the bioactive compounds and their biological activities, the results show that the antioxidant activity was significantly correlated with the TPC, TFC, and TPrC (Fig 1), revealing that the three groups of bioactive compounds promoted antioxidant activity, which is consistent with previous research results [45]. TPC, TFC, and TPrC had a certain inhibitory effect on BChE, while TPrC, TAC, TChC, and TCaC promoted on the inhibition of AChE. All the bioactive compounds detected in this study contributed to the anti-HepG2 cell proliferation due to their remarkable correlation, which indicates that *E*. *mollis* leaf extract could be exploitable in the fields of food, healthcare, and medical care. Interestingly, TAC and TCaC were negatively correlated with antioxidant activity. This could be because the extracts used to evaluate the biological activity were complex mixtures in which several compounds can inevitably react with each other, finally leading to an underestimation of the antioxidant activity values of TAC and TCaC [46]. Moreover, previous studies have proven that inhibition of AChE and BChE in plant extracts is mainly attributed to the natural alkaloids contained in the extracts [47], but in the current study, TAC had a more noticeable effect on the inhibition of AChE than BChE, which needs further exploration. These results indicate that the different kinds of bioactive compounds present in the leaf extracts might contribute to the biological activity.

### HPLC-ESI-Q TRAP-MS/MS analysis

**Fig 2:**
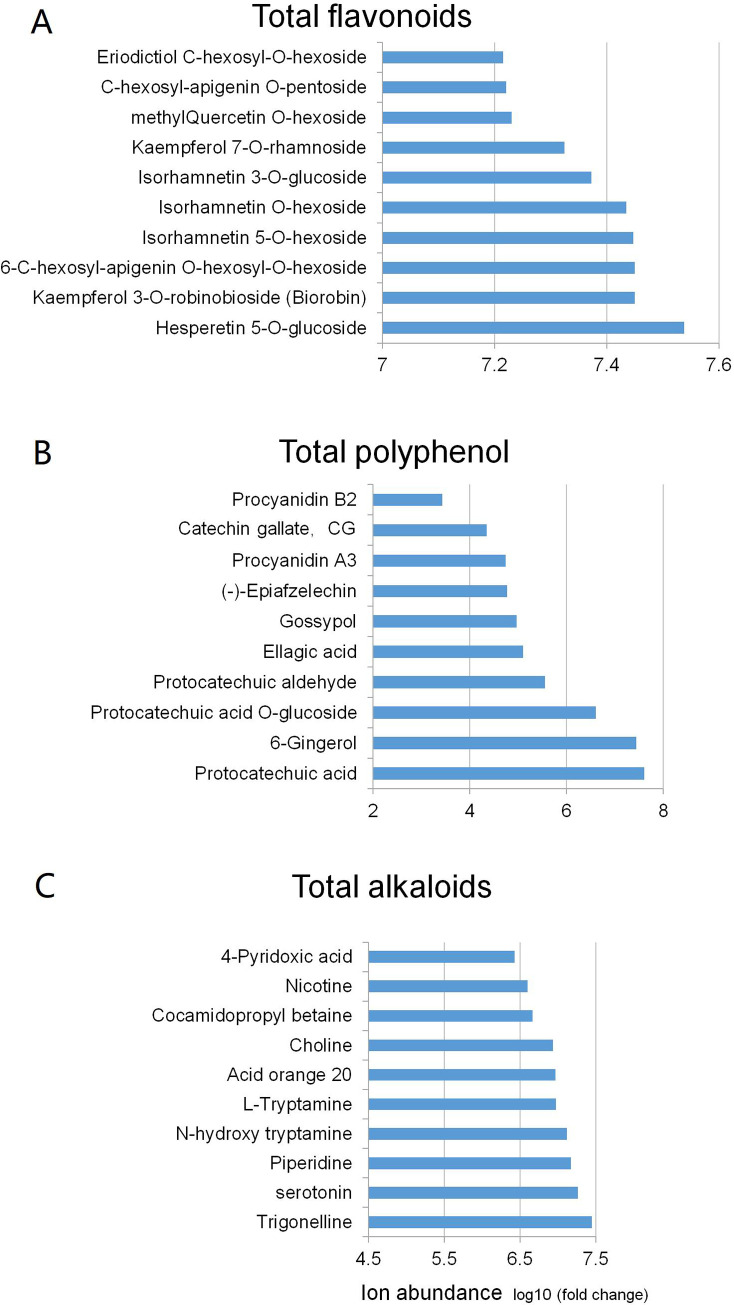
Metabolic profiling in tested *E*. *mollis* samples. (A) Top 10 most abundant components in the class of total flavonoids; (B) Top 10 most abundant components in the class of total phenolics; (C) Top 10 most abundant components in the class of total alkaloids.

In view of the above research results, *E*. *mollis* leaf extracts are rich in bioactive compounds, which mainly contribute to the biological activity. A total of 1,017 bioactive compounds were detected in the leaves of *E*. *mollis* using the widely targeted metabolomics approach which provides new information on the potential use of the leaves for further research. The detailed quantification and the ion abundance of all detected components are presented in S1 Table. The detected components could be classified into 23 classes, predominantly organic acids and derivatives, flavone, amino acid and derivatives, lipids, and phenylpropanoids (Table 6). In contrast, the content of quinones, sterides, proanthocyanidins were found to be very low in the leaves of *E*. *mollis*. In the present study, we quantitatively determined the total content of flavonoids, phenolics, alkaloids, procyanidins, chlorophyll and carotene. Except for chlorophyll and carotene, which were not detected due to the unsuitable method [48]. Mapping in the 1,017 components, the other bioactive compounds are divided into three classes: total flavonoids (including flavone, flavonol, flavonoid, flavanone, anthocyanins, and isoflavone), total phenolics (including polyphenols, and proanthocyanidins), and total alkaloids. And the top 10 most abundant components found in each class are shown in Fig 2.

**Table 6:** Classification of the identified bioactive components in the leaves extracts of *E*.*mollis*.

Class	Number of Compounds	Class	Number of Compounds
Organic acids and derivatives	141	Vitamins and derivatives	26
Flavone	124	Carbohydrates	24
Amino acid and derivatives	103	Phenolamides	23
Lipids	87	Alcohols	22
Phenylpropanoids	86	Anthocyanins	20
Alkaloids	67	Polyphenol	20
Nucleotide and derivates	64	Indole derivatives	10
Flavonol	42	Isoflavone	10
Others	42	Quinones	7
Flavonoid	32	Sterides	6
Terpene	32	Proanthocyanidins	3
Flavanone	26		

The bioactive components found in *E*. *mollis* leaves may have potential biological activity. In particular, the total flavonoids, which constitute 24% of *E*. *mollis* leaves, could play important roles in the protection against free radical cell damage and several cancers [45]. Previous studies have proven that proanthocyanidins can promote the antioxidant activity [21]. Moreover, phenolics are not only antioxidants but have also been proven to be closely related to some anticancer activities [49]. Similarly, alkaloids have a potential effect on the prevention of some cancers and inhibition of ChE [47, 50]. In addition, the leaves of *E*. *mollis* are rich in organic acids, vitamins, amino acids, lipids and other nutrients. For these reasons, the bioactive components of *E*. *mollis* leaves reported in present study may provide useful information for further related research.

## Conclusions

The results of the current investigation reveal that the leaves of *E*. *mollis* contain abundant functional components with different biological activities. Furthermore, there were significant differences in the extraction yield, bioactive compound content, antioxidant activity, ChE inhibition, and anti-HepG2 cell proliferation of *E*. *mollis* leaf extracts from different extraction solvents. Therefore, the appropriate solvent should be selected to meet a specific demand. In particular, the water leaf extracts exhibited the highest activity levels in the antioxidant assays while the methanol extracts exhibited the best ChE inhibition and anti-HepG2 cell proliferation activity. According to the correlation analysis, these biological activities are closely related to the total phenolic, flavonoid, and proanthocyanidin contents. In addition, due to the narrow distribution of *E*. *mollis*, the variation in environmental conditions was limited, and so the effect of different habitats was lower than the effects of different solvents. In conclusion, the methanol and water extracts of *E*. *mollis* could be applied in the fields of healthcare, pharmaceuticals, and food processing.

## Supporting information

S1 Table List of the components detected in the leaf from *E*. *mollis*. (XLSX)
